# Deoxyribonuclease 1-Mediated Clearance of Circulating Chromatin Prevents From Immune Cell Activation and Pro-inflammatory Cytokine Production, a Phenomenon Amplified by Low Trap1 Activity: Consequences for Systemic Lupus Erythematosus

**DOI:** 10.3389/fimmu.2021.613597

**Published:** 2021-03-04

**Authors:** Jasmin Felux, Annika Erbacher, Magali Breckler, Roxane Hervé, Delphine Lemeiter, Hans Georg Mannherz, Markus Napirei, Hans-Georg Rammensee, Patrice Decker

**Affiliations:** ^1^Department of Immunology, Institute for Cell Biology, University of Tübingen, Tübingen, Germany; ^2^Li2P, University Sorbonne Paris Nord, Bobigny, France; ^3^INSERM UMR 1125, Bobigny, France; ^4^Department of Anatomy and Molecular Embryology, Medical Faculty, Ruhr-University Bochum, Bochum, Germany

**Keywords:** DNase1, TRAP1, extracellular chromatin, clearance, inflammation, lupus, autoimmunity

## Abstract

Increased concentrations of circulating chromatin, especially oligo-nucleosomes, are observed in sepsis, cancer and some inflammatory autoimmune diseases like systemic lupus erythematosus (SLE). In SLE, circulating nucleosomes mainly result from increased apoptosis and decreased clearance of apoptotic cells. Once released, nucleosomes behave both as an autoantigen and as a damage-associated molecular pattern (DAMP) by activating several immune cells, especially pro-inflammatory cells. Deoxyribonuclease 1 (DNase1) is a major serum nuclease whose activity is decreased in mouse and human lupus. Likewise, the mitochondrial chaperone tumor necrosis factor (TNF) receptor-associated protein-1 (Trap1) protects against oxidative stress, which is increased in SLE. Here, using wild type, DNase1-deficient and DNase1/Trap1-deficient mice, we demonstrate that DNase1 is a major serum nuclease involved in chromatin degradation, especially when the plasminogen system is activated. *In vitro* degradation assays show that chromatin digestion is strongly impaired in serum from DNase1/Trap1-deficient mice as compared to wild type mice. *In vivo*, after injection of purified chromatin, clearance of circulating chromatin is delayed in DNase1/Trap1-deficient mice in comparison to wild type mice. Since defective chromatin clearance may lead to chromatin deposition in tissues and subsequent immune cell activation, spleen cells were stimulated *in vitro* with chromatin. Splenocytes were activated by chromatin, as shown by interleukin (IL)-12 secretion and CD69 up-regulation. Moreover, cell activation was exacerbated when Trap1 is deficient. Importantly, we also show that cytokines involved in lupus pathogenesis down-regulate Trap1 expression in splenocytes. Therefore, combined low activities of both DNase1 and Trap1 lead to an impaired degradation of chromatin *in vitro*, delayed chromatin clearance *in vivo* and enhanced activation of immune cells. This situation may be encountered especially, but not exclusively, in SLE by the negative action of cytokines on Trap1 expression.

## Introduction

Increased concentrations of circulating chromatin are observed in some inflammatory autoimmune diseases, such as systemic lupus erythematosus (SLE) and rheumatoid arthritis, as well as in sepsis and cancers. SLE is a rheumatic disease of unknown etiology affecting mainly women and leading to skin, kidney, joint, nervous system and cardiovascular manifestations. SLE is characterized by the production of autoantibodies directed mostly against nuclear autoantigens. In SLE, impaired clearance of chromatin leads to accumulation of plasma mono- and oligo-nucleosomes (1–3). The nucleosome is a complex composed of 180 base pairs of DNA, one molecule of histone H1 and two molecules of histones H2A, H2B, H3, and H4. These nucleosomes participate to lupus pathogenesis at two levels. On the one hand, they represent a major lupus autoantigen. IgG3 anti-nucleosome autoantibodies are associated with active disease (4) and nucleosome-specific T-cells are present in patients (5). On the other hand, extracellular nucleosomes behave as a damage-associated molecular pattern (DAMP) and are pro-inflammatory. They induce activation of dendritic cells (6) and polymorphonuclear neutrophils (PMN) (7), leading to secretion of the key lupus cytokine interferon (IFN)-α (8) as well as pro-inflammatory cytokines (9) and the release of soluble CEACAM8 (10). This was observed in mice and humans, both in healthy individuals and lupus patients, indicating that those cells have the capacity to respond to nucleosomes but that the key pathogenic event is an elevated nucleosome concentration in patients only. The release of chromatin is believed to trigger the production of the anti-chromatin autoantibody family in SLE, namely anti-double-stranded DNA (a lupus marker), anti-histones and nucleosome-restricted autoantibodies. Nucleosomes were also shown to bind laminin β1, which is aberrantly expressed in the glomerular basement membrane during lupus nephritis (11).

Deoxyribonuclease 1 (DNase1) is a major nuclease in body fluids and is thought to play an important role in degrading chromatin, and especially in removing DNA from nuclear autoantigens, at sites of high cell turnover, i.e., cell death (12, 13). DNase1 is known to degrade isolated double-stranded DNA in a Ca^2+^ and Mg^2+^ dependent manner but its activity on circulating chromatin-organized DNA (where DNA is protected by histones) is less clear. For example, DNase1 is also involved in internucleosomal cleavage of chromatin *in vitro* but to which extent DNase1 participates in nucleosomal DNA degradation in body fluids and in cooperation with which cofactors requires further investigations. However, DNase1 activity is decreased in SLE patients and inversely correlates with disease activity, e.g., lupus nephritis (14) or levels of anti-nucleosome autoantibodies (15). Moreover, DNase1 mutations were detected in SLE and were associated with high serum titres of anti-DNA autoantibodies (16, 17). In addition, DNase1 inhibitors are involved in its low activity in patients. A low serum concentration of DNase1 correlated with high concentrations of the DNase inhibitor actin in a mouse model of SLE (18). Furthermore, raised serum levels of the DNase1 inhibitor were associated with the presence of anti-nuclear autoantibodies in humans (19). Additionally, DNase inhibitory antibodies were detected in sera of lupus patients that could cause decreased DNase1 activities (20). The link between DNase1 and SLE development is further supported by studies in mice. DNase1-deficient mice on a lupus-prone genetic background develop a SLE-like disease with production of anti-nuclear antibodies, immune complex deposition in kidneys leading to glomerulonephritis (21).

The former *DNase1*-knockout (KO) mice originally described by Napirei et al. carry an additional mutation in the gene coding for tumor necrosis factor (TNF) receptor-associated protein-1 (Trap1)/heat shock protein 75 (Hsp75) which is located at the opposite DNA strand to the *DNase1* gene. These mice possess a combined *DNase1*^−/−^*/Trap1*^*m*/*m*^ genotype and are deficient for both DNase1 and Trap1 (22). Nevertheless, development of a lupus-like disease has been recently confirmed in a different strain of DNase1-deficient mice with an intact *Trap1* gene (23).

TRAP1, a member of the HSP90 family, is a mitochondrial chaperone that regulates stress responses (24). As Trap1 is not secreted and has no DNase activity, *DNase1*^−/−^*/Trap1*^*m*/*m*^ mice can be used for studying serum DNase1 function. Interestingly, Trap1 protects against oxidative stress, which is increased in SLE. Actually, Trap1deficiency leads to enhanced oxidative stress in mice (25). Likewise, Trap1 acts as an anti-apoptotic/survival protein, whereas increased apoptosis has been reported in SLE. Thus, Trap1 activity or expression might be transiently altered in SLE, maybe only in some organs. Actually, *Trap1* gene is silenced in end-stage lupus disease, at least in the kidneys (26). In addition, although the consequences on Trap1 biological activity are unknown, *Trap1* gene polymorphisms associated with susceptibility to SLE and efficacy of glucocorticoids have been reported, especially in immune cells (27).

Altogether, those data indicate that impaired clearance of chromatin is pathogenic due to loss of tolerance and suggest that low DNase1 activity is involved in lupus pathogenesis by favoring anti-chromatin autoantibody production. Nevertheless, DNase1 is not the only nuclease of the blood stream and not the only one potentially altered in SLE. For example, loss of function of DNase1-like 3 causes aggressive SLE (28). This enzyme digests chromatin in microparticles released from apoptotic cells and DNase1-like 3-deficient mice also develop a SLE-like disease (29). To better understand the protective role of DNase1 against SLE and its contribution to degradation of circulating chromatin, we first analyzed the degradation of chromatin *in vitro* in serum from wild type and DNase1-deficient mice (using *DNase1*^−/−^*/Trap1*^*m*/*m*^ mice). We next analyzed the *in vivo* clearance of chromatin in those mice. Because part of circulating chromatin deposits in the spleen in mice (30), we investigated the consequences of chromatin on immune cell activation in this organ of wild type, *DNase1*^−/−^*/Trap1*^*m*/*m*^ and DNase1-deficient (*DNase1*^*lacZ*/*lacZ*^) reporter mice without Trap1 impairment. Finally, we measured *ex vivo* Trap1 expression by splenocytes from the three mouse strains and we determined the *in vitro* effects of cytokines on Trap1 expression by splenocytes.

## Methods

### Mice

Generation of *DNase1*^−/−^*/Trap1*^*m*/*m*^ mice has been previously described (21). Primarily published as DNase1-KO, this mouse strain harbors an aberrant *Trap1* mutation (*Trap1*^*m*/*m*^), which leads to a lack of Trap1m protein within the detection limit of a Western Blot (22). Mice used in this study were on a pure C57BL/6 genetic background and do not develop a lupus-like disease. *DNase1*^*lacZ*/*lacZ*^ KO/reporter mice on a C57BL/6 genetic background [DNase1^tm1(KOMP)Vlcg^, Mouse Genome Informatics (MGI): 3813105] were obtained from the Canadian Mouse Mutant Repository (CMMR, Hospital for Sick Children, Toronto, Canada). Mice carrying the C1qa-KO were generated by Marina Botto (31). They have been backcrossed on a C57BL/6 genetic background for ten generations and then crossed with *DNase1*^−/−^*/Trap1*^*m*/*m*^ mice to generate *DNase1*^−/−^*/Trap1*^*m*/*m*^*/C1qa*^−/−^ mice. Wild type C57BL/6 mice (Charles River Laboratories, Sulzfeld, Germany) were used as controls. Toll-like receptor (TLR) 2/TLR4-deficient mice (TLR2-KO mice (32) on a C3H/HeJ genetic background) were obtained from Hermann Wagner (Munich, Germany). All mouse strains compared in this study were housed in the same animal facility. Mouse experiments had been approved by the local ethics committee (Regierungspräsidium Tübingen, reference IM4/07 and the Darwin Committee of the University Sorbonne Paris Nord).

### Chromatin Purification

Oligo- and mono-nucleosomes were purified from calf thymus under sterile conditions as previously described (6, 7, 33). Briefly, nuclei were isolated, then digested with micrococcal nuclease (Sigma-Aldrich) and lysed. After centrifugation, supernatants containing chromatin fragments of different sizes were collected and further purified by ultracentrifugation on 5–29% sucrose gradients. As a control, an empty sucrose gradient was loaded with lysis buffer only. After centrifugation, several fractions were collected for both the chromatin-loaded and the buffer-loaded gradients. The latter was used as a negative control in cell culture (nucleosome purification buffer). All chromatin fractions were analyzed by agarose gel electrophoresis (1.5%) and SDS-PAGE (18%). Fractions containing either oligo-nucleosomes or mono-nucleosomes were harvested. To prepare nucleosomes depleted of histone H1, chromatin was incubated in 0.5 M NaCl, 10 mM Tris pH 7.4, 0.2 mM EDTA (final concentration) for 20 min at 4°C prior to ultracentrifugation on sucrose gradient containing 0.5 M NaCl. Representative preparations used in the present study are shown in Supplementary Figure 1. Of note, free self DNA is not strongly immunogenic and core histones are 100 % conserved between mouse and calf. Without indication, chromatin refers to H1-containing nucleosomes.

### *In vitro* Chromatin Degradation Assays

H1-depleted oligo-nucleosomes (37.5–150 μg/ml) were incubated at 37°C for different time points with/without 250 units/ml heparin in serum (5%) diluted in reaction buffer (10 mM Hepes, 50 mM NaCl, 2 mM MgCl_2_, 2 mM CaCl_2_, 40 mM β-glycerophosphate, final concentrations). The reaction was stopped with 60 mM EDTA (final concentration). Samples were then analyzed by agarose gel (1.5%) electrophoresis.

### *In vivo* Chromatin Clearance

Oligo-nucleosomes (50 μg) were diluted in DPBS (100 μl final volume) and were intravenously injected in different mouse strains via the tail vein. Mice were bled by retro-orbital puncture at 2 min post injection (injection control and “100%” start value) and at varying time points up to 4 h. Plasma was prepared using BD Microtainer® tubes. The chromatin fractions were isolated out of 40 μl plasma using the NucleoSpin® PlasmaXS Kit (Macherey-Nagel) which allows the extraction of very small DNA fragments. The isolated DNA was analyzed on 1.5% agarose gels and visualized by ethidium bromide staining. Pictures were taken at different exposure times to evaluate low as well as high signals. The samples were quantitated by measuring the mean intensities and pixels of each signal. The degradation state of chromatin after different time points was displayed as percentage of the starting value of each individual mouse. Results are presented as mean ± standard error of the mean (SEM) of all mice.

### Cell Culture

Spleen leukocytes were prepared using a 70 μm cell strainer and red blood cells were lysed in cold hypotonic buffer (NH_4_Cl, KHCO_3_, EDTA). Cells (1.5 × 10^6^/ml) were cultured in X-VIVO 15 medium (Lonza) without serum in medium only or supplemented with mono-nucleosomes (10–50 μg/ml), its purification buffer (gradient), lipopolysaccharides (LPS, from *S. typhimurium*, 1 μg/ml, with heat-inactivated fetal calf serum, Sigma-Aldrich) or synthetic unmethylated CpG motif-containing oligonucleotides (CpG-ODN, 2 μM, InvivoGen). Cell culture supernatants were collected at 24 h and cells were harvested after 65 h to estimate cell activation by ELISA and flow cytometry. Alternatively, spleen cells (2 × 10^6^/ml) were cultured for 20 h in medium alone or supplemented with IFN-γ (400 ng/ml) or interleukin (IL)-6 (100 ng/ml) and Trap1 expression was analyzed by Western-blot.

### Flow Cytometry

Spleen cells were either analyzed *ex vivo* or after 65 h in culture to determine the phenotype or estimate cell activation, respectively. Cells were stained with monoclonal antibodies specific for CD3 (FITC- or PerCP-Cy5.5-conjugated, clone 17A2) or CD4 (Alexa Fluor 488-conjugated, clone RM4-5), CD8a (BV510-conjugated, clone 53-6.7), CD45R/B220 (PE-conjugated, clone RA3-6B2), CD19 (APC-conjugated, clone 1D3), CD11c (PE-conjugated, clone HL3), CD86 (PE-conjugated, clone GL1), CD69 (PE-Cy7-conjugated or biotin-conjugated, clone H1.2F3, followed by PE- or APC-conjugated streptavidin) or the corresponding isotype control, at 4°C in staining buffer [PBS containing 5% heat-inactivated FCS, 100 μg/ml human γ-globulin (Calbiochem), 0.02% sodium azide] and according to classical protocols. All antibodies were purchased from BD Biosciences (except anti-CD8, BioLegend). Cell viability was estimated by propidium iodide (PI) staining. Spleen cell activation was estimated by CD69 expression on total living cells gated according to size and granularity and exclusion of dead (PI-positive) cells. Cells were analyzed on a four-color FACSCalibur or an eight-color FACSCanto II apparatus (Becton Dickinson). Data were evaluated with CellQuest Pro, FACS Diva or FlowJo software (BD Biosciences). Results are presented as mean fluorescence intensity (MFI) or percentages of cells positive for the indicated markers among all living cells. Data are depicted as mean ± standard deviation (SD) of triplicates in representative experiments or as mean ± SEM in pooled data.

### ELISA

Cytokine secretion by splenocytes was measured in culture supernatants after 24 h. IL-12p40/p70 was quantified by sandwich ELISA using monoclonal antibody pair and streptavidin-peroxidase conjugate (BD Biosciences) according to the manufacturer's instructions. Concentrations in cell culture supernatants are depicted as mean ± SD of triplicates in representative experiments or as mean ± SEM in pooled data.

### Protein Extracts and Western Blot

Spleen cells (*ex vivo* or after cell culture) were lysed in RIPA buffer on ice. After sonication, cell debris was sedimented at 10,000 g for 10 min and the protein content of the supernatant was estimated by the Bradford protein assay. Equal amounts of total proteins from cell extracts (25 or 50 μg, denatured at 95°C for 5 min) or recombinant Trap1 (400 ng, Sigma-Aldrich) were separated by SDS-PAGE, either on 4% (v/v) concentrating and 10% (v/v) resolving gels, or on 4–15% stain free gradient gels (Miniprotean® TGX stain free protein gels, Bio-Rad). Electrophoresis was carried out at 80–120 V using standard Tris-glycine SDS running buffer and then semidry blotting (PerfectBlue™, PeqLab) using a nitrocellulose membrane (Macherey-Nagel) and Towbin buffer was performed. Alternatively, samples were electrotransferred onto PVDF membrane (Trans-Blot Turbo Mini, 0.2 μm PVDF Transfer Packs, Bio-Rad) with Trans-Blot Turbo system (Bio-Rad). Blots were incubated in standard TBS-Tween buffer [0.05% (v/v) Tween 20] supplemented with 5% (w/v) skimmed milk powder for 2 h at room temperature (RT) prior to addition of the primary antibodies (mouse anti-β-actin, 1:5.000, #A1978, Sigma-Aldrich or 1:250, #sc-47778, Santa Cruz; mouse anti-human/mouse TRAP1, 1:1,500, #612344 and mouse anti-human/mouse Hsp60, 1:1,500, #611562, BD Transduction Laboratories™). Incubation with the primary antibodies was performed over night at 4°C. Afterwards, the blots were repeatedly washed with TBS-Tween buffer, incubated with the secondary antibody (anti-mouse IgG conjugated to HRP, 1:3,000, #315-035-008 or #115-035-062, 1:4,000, Jackson ImmunoResearch) for 1 h at RT and washed again. Chemiluminescence detection was performed with appropriate reagents from Bio-Rad Laboratories and the blots were analyzed using the ChemiDoc™ System with the ImageLab 5.0 software (Bio-Rad Laboratories). For quantification, densitometric analysis was performed on saved pictures using ImageLab software and the Trap1 signal was normalized to the corresponding β-actin signal in each individual sample.

### Statistical Analysis

In all figures, the legend indicates whether representative experiments and/or pooled data are shown, the number of independent experiments, the number of mice used and the number of independent chromatin preparations. *In vivo* nucleosome clearance in wild type and *DNase1*^−/−^*/Trap1*^*m*/*m*^ mice was compared using two-tailed unpaired *t*-test without Welch's correction after having checked that both groups follow a Gaussian distribution and have similar variances. Concentrations of IL-12 in culture supernatants from wild type and *DNase1*^−/−^*/Trap1*^*m*/*m*^ splenocytes were compared using two-tailed Wilcoxon matched-pairs signed rank test. CD69 expression (MFI and percentages of positive cells) by cultured wild type and *DNase1*^−/−^*/Trap1*^*m*/*m*^ splenocytes were compared using two-tailed paired *t*-test or two-tailed Wilcoxon matched-pairs signed rank test. CD69 MFI on *DNase1*^−/−^*/Trap1*^*m*/*m*^ splenocytes vs. wild type or *DNase1*^*lacZ*/*lacZ*^ splenocytes was compared using two-tailed Mann Whitney test or two-tailed unpaired *t*-test with Welch's correction. *Ex vivo* Trap1/β-actin expression ratios by wild type and *DNase1*^*lacZ*/*lacZ*^ splenocytes were compared using two-tailed Mann Whitney test. Trap1/β-actin expression ratios by splenocytes cultured with/without IFN-γ or IL-6 were compared using two-tailed Wilcoxon matched-pairs signed rank test. Data were analyzed using GraphPad Prism software (*p* ≤ 0.05 was considered significant).

## Results

### *In vitro* Chromatin Degradation Is Dramatically Impaired in Serum From DNase1-Deficient Mice

**Figure 1:**
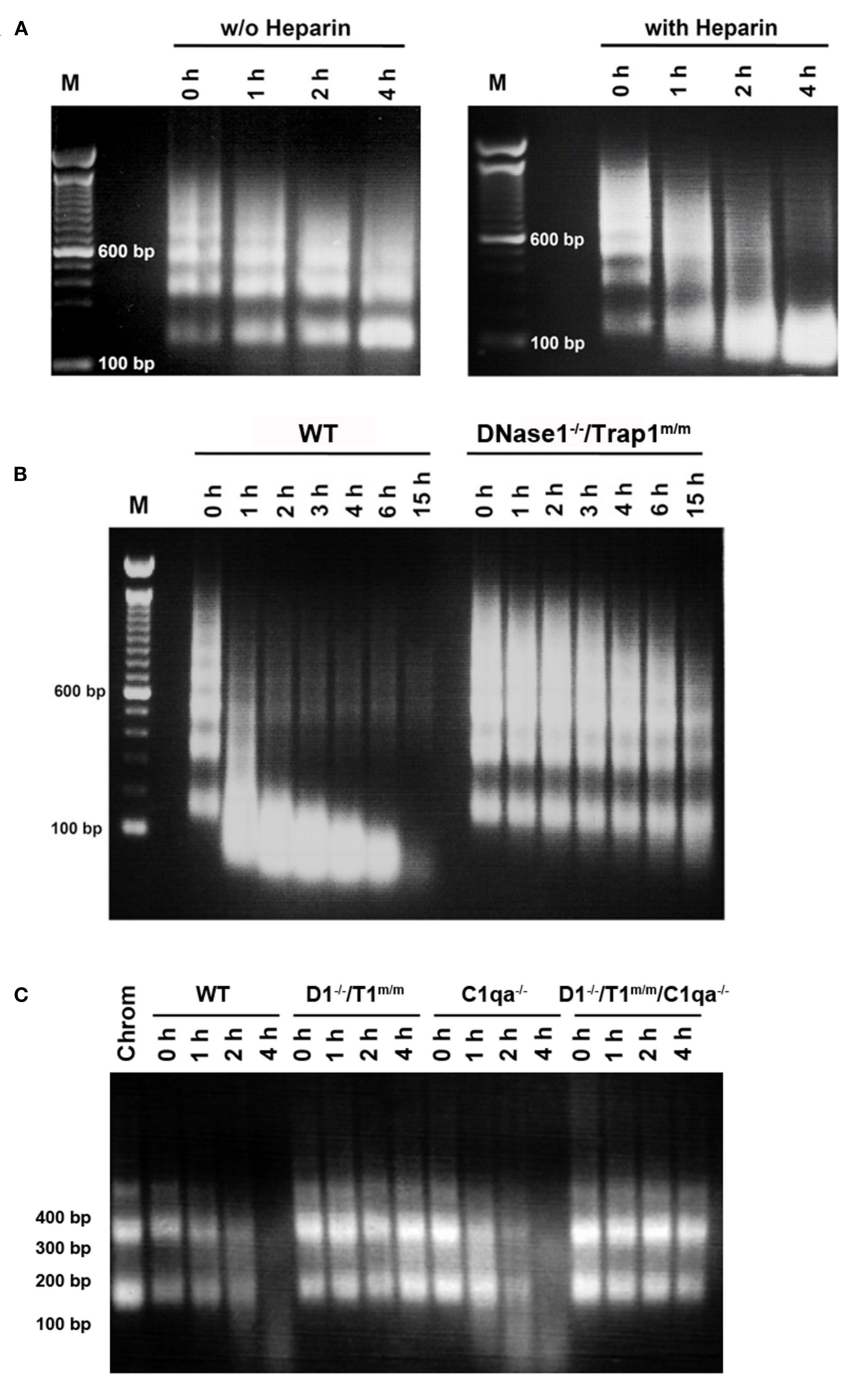
DNase 1 is the major serum nuclease degrading chromatin. **(A)** Heparin enhances chromatin degradation in serum. Normal serum of a wild type C57BL/6 mouse was incubated in the presence of purified chromatin for 0–4 hours (h) with (right) or without (w/o, left) heparin. Chromatin was then analyzed by 1.5% agarose gel electrophoresis. One representative of five independent experiments is depicted. bp, base pair; M, molecular weight marker (100 bp ladder). **(B)** Chromatin degradation is impaired in serum from DNase1-deficient mice. Sera from wild type (WT) or *DNase1*^−/−^*/Trap1*^*m*/*m*^ mice were incubated with chromatin in the presence of heparin for 0–15 h. Chromatin was then analyzed by 1.5% agarose gel electrophoresis. One representative of nine independent experiments is depicted. **(C)** C1q is not required for optimal chromatin degradation. Sera from wild type (WT), *DNase1*^−/−^*/Trap1*^*m*/*m*^ (D1^−/–^/T1^m/m^), *C1qa*^−/−^ or *DNase1*^−/−^*/Trap1*^*m*/*m*^*/C1qa*^−/−^ (D1^−/–^/T1^m/m^/C1qa^−/–^) mice were incubated with chromatin in the presence of heparin for 0–4 h. Chromatin was then analyzed by 1.5% agarose gel electrophoresis. One representative of three independent experiments is depicted (using three independent chromatin preparations). For comparison, purified chromatin (Chrom) not incubated with serum was loaded.

Chromatin degradation was analyzed in physiological conditions, using serum to mimic circulating chromatin, instead of purified DNase1, as other serum proteins may influence degradation. Chromatin degradation assays were performed and compared in the presence or absence of heparin. Indeed, chromatin degradation by serum DNase1 is enhanced by heparin (34) as well as by the plasminogen system (35), which both remove DNA-associated proteins and thus renders DNA accessible for DNase1. In the latter study, heparin has been shown to enhance DNA degradation by serum in hydrogen peroxide-treated necrotic cells, but was not tested on purified oligo-nucleosomes. Actually, heparin also supports the activity of the plasminogen system (36), the activity of which is reduced in SLE, resulting in impaired fibrinolysis and potentially leading to thrombosis, a frequent lupus manifestation (37). Moreover, it is a DNase1-like 3 inhibitor (34). Thus, the use of heparin allows focusing on DNase1 in whole serum. Heparin is also naturally produced by mast cells and released in the circulation at sites of tissue injury. In our assays, oligo-nucleosomes are slightly degraded in wild type serum, indicating the presence of active nucleases (Figure 1A left, without heparin). Nevertheless, chromatin is rather resistant to complete degradation. By comparing wild type serum supplemented or not with heparin, we could show that heparin enhances chromatin degradation (Figure 1A), as evidenced by a faster kinetics and the degradation pattern. With heparin, oligo-nucleosomes are completely degraded at 4 h. Moreover, instead of individual bands, a DNA smear is visible as a result of random cleavage. DNA is degraded to fragments smaller than 146 bp, the size of core nucleosomal DNA. Without heparin, internucleosomal cleavage occurs, yielding accumulation of mono-nucleosomes. These results suggest that the cooperation between DNase1 and proteases naturally present in the serum is involved in the efficient degradation of chromatin. DNase1-like 3 however also participates to the process. Using these settings (evaluating DNase1 activity in the presence of heparin), we next compared chromatin degradation by serum from wild type C57BL/6 mice or DNase1-deficient mice. Because Trap1 has no nuclease activity and is located in mitochondria but is not secreted, we used here *DNase1*^−/−^*/Trap1*^*m*/*m*^ mice. As compared with serum from wild type mice, chromatin degradation is strongly impaired in serum from *DNase1*^−/−^*/Trap1*^*m*/*m*^ mice (Figure 1B). Both the kinetics and the degradation pattern are affected. Indeed, after 15 h, chromatin is completely degraded in wild type serum whereas it is still clearly detectable in serum of *DNase1*^−/−^*/Trap1*^*m*/*m*^ mice. Likewise, after 1 h, chromatin was massively degraded by wild type serum, whereas degradation was hardly visible in DNase1-deficient serum. Moreover, internucleosomal cleavage (although low) is mainly observed in DNase1-deficient serum, with degradation of oligo-nucleosomes and a slight accumulation of mono-nucleosomes, whereas it switches to random degradation in wild type serum, generating fragments smaller than core particle DNA and finally resulting even in total degradation. These results show that DNase1 is the major serum nuclease degrading chromatin. Because C1q was reported to enhance chromatin degradation in human necrotic lymphocytes (38), we compared then chromatin degradation in sera from wild type, *DNase1*^−/−^*/Trap1*^*m*/*m*^, *C1qa*^−/−^ (which possess a normal DNase1 activity) and *DNase1*^−/−^*/Trap1*^*m*/*m*^*/C1qa*^−/−^ mice. As shown in Figure 1C, chromatin degradation was not altered in C1q-deficient serum as compared to wild type serum, indicating that C1q is not required for optimal degradation of isolated chromatin. Nearly no degradation was observed in sera from both *DNase1*^−/−^*/Trap1*^*m*/*m*^ and *DNase1*^−/−^*/Trap1*^*m*/*m*^*/C1qa*^−/−^ mice, yielding a similar pattern. Chromatin degradation in serum occurs thus in a DNase1-dependent but C1q-independent manner.

### Chromatin Clearance Is Delayed *in vivo* in DNase1-Deficient Mice

**Figure 2:**
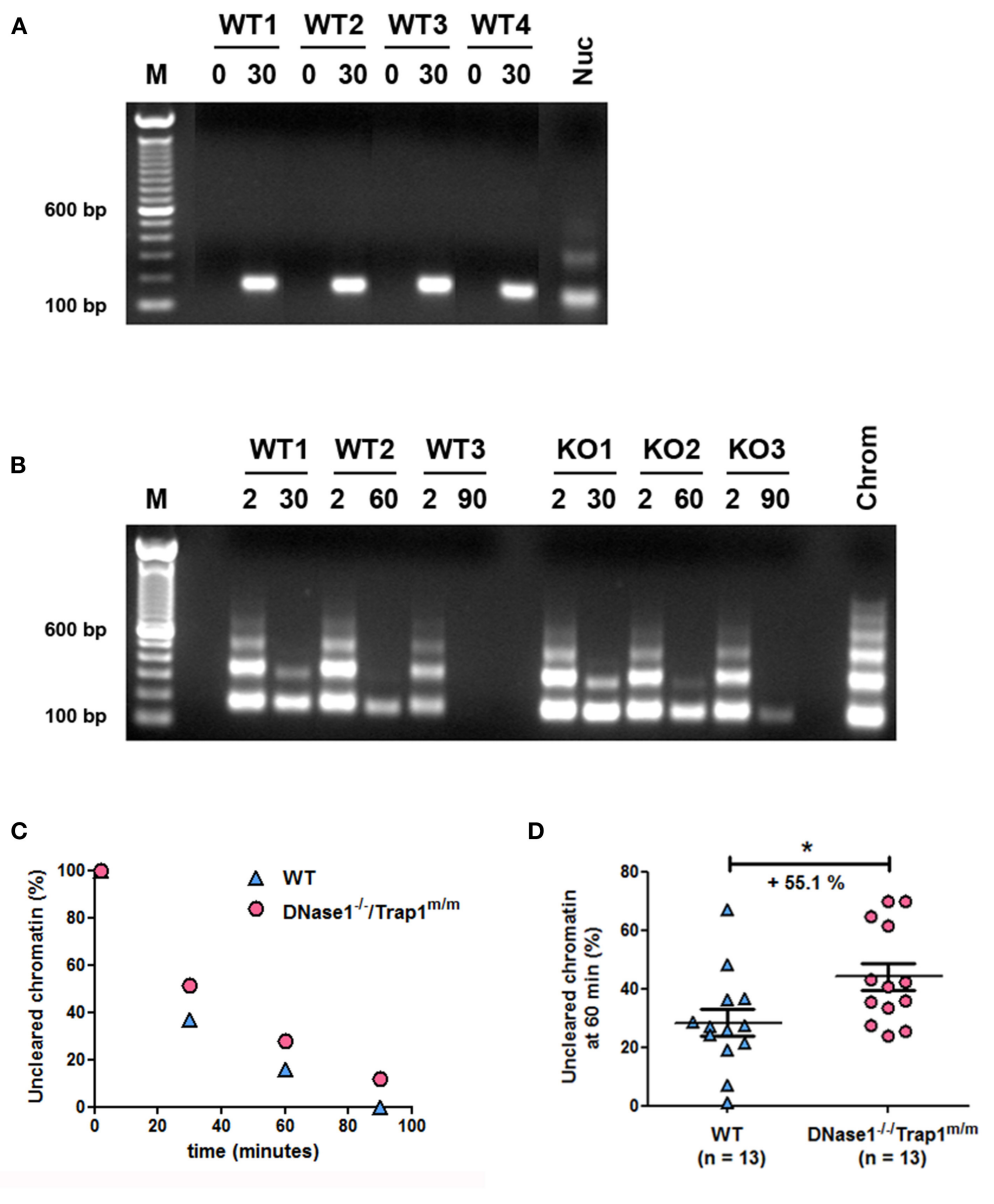
*In vivo* chromatin clearance is delayed in DNase1-deficient mice. **(A)** Chromatin is recovered in plasma from chromatin-injected mice. Four wild type (WT) mice were injected intravenously with purified mono-/di-nucleosomes. Plasma was prepared from tested mice before (0) and 30 min after injection. DNA was isolated from individual mice and analyzed by agarose gel electrophoresis. For comparison, purified nucleosomes (Nuc) used for injections were loaded. One representative of four independent experiments is depicted. The different lanes shown were extracted from the same gel without modification of brightness. bp, base pair; M, molecular weight marker (100 bp ladder). **(B)** Kinetics of chromatin clearance in three wild type and three DNase1-deficient [KO (knockout), *namely DNase1*^−/−^*/Trap1*^*m*/*m*^] mice. Mice were injected intravenously with purified oligo-nucleosomes and plasmas were prepared 2 and 30, 2 and 60, or 2 and 90 min post injection. One mouse was used for one time point (thus, each mouse was bled two times post injection). DNA was isolated and analyzed as in **(A)**. For comparison, purified chromatin (chrom) used for injections was loaded. Shown is one representative experiment of three independent experiments, each with six mice (three mice per group). **(C)** DNA isolated from plasma in **(B)** was quantified by measuring mean intensities and pixels of each signal on pictures from agarose gels and normalized to the 2-min time point for each mouse; Wild type (*n* = 3, blue triangles) and *DNase1*^−/−^*/Trap1*^*m*/*m*^ (*n* = 3, pink circles) mice are depicted, with one mouse per time point in each group. **(D)** Clearance of circulating chromatin was analyzed as in **(B,C)** at 60 min post chromatin injection in 26 independent mice, both in wild type (*n* = 13, blue triangles) and *DNase1*^−/−^*/Trap1*^*m*/*m*^ (*n* = 13, pink circles) mice. For each mouse, the 60-min signal was normalized to the 2-min signal. Shown are data pooled from four independent experiments, with three independent chromatin preparations. Mean and SEM are depicted. *p < 0.05 (two-tailed unpaired *t*-test).

To support a physiological role of DNase1 in the elimination of circulating chromatin, we analyzed chromatin degradation *in vivo*. Mice were intravenously injected with purified oligo-nucleosomes and chromatin was consecutively followed in plasma. First, we set up our system with mono-/di-nucleosomes to prove that injected chromatin is detectable in plasma. DNA was isolated from plasma collected by retro-orbital punctation and analyzed by agarose gel electrophoresis. Whereas no circulating DNA was observed in plasma of wild type mice before injection, circulating nucleosomal DNA was detected 30 min post injection and in all tested mice (Figure 2A). With these settings, we analyzed and compared the kinetics of chromatin clearance in wild type and *DNase1*^−/−^*/Trap1*^*m*/*m*^ mice. Three wild type and three DNase1-deficient mice were intravenously injected with purified oligo-nucleosomes and nucleosomal DNA was estimated after 2 and 30, 2 and 60, or 2 and 90 min. For each mouse, the 2-min time point is used as a reference. In both wild type and DNase1-deficient mice, chromatin is cleared in a time-dependent manner, with progressive elimination of oligo-nucleosomes and then mono-nucleosomes (Figure 2B). However, chromatin clearance is more effective in wild type mice, as shown by the complete elimination of circulating chromatin after 90 min in contrast to DNase1-deficient mice. DNA isolated from plasma was then quantified and normalized to the 2-min time point for each mouse (Figure 2C). The data confirm both time-dependent clearance of chromatin *in vivo* and delayed clearance in DNase1-deficient mice. To precisely determine and compare *in vivo* chromatin clearance in wild type and *DNase1*^−/−^*/Trap1*^*m*/*m*^ mice, this experiment was reproduced in 26 mice, focusing on the 60-min time point (with 2-min time point as internal reference for each mouse). As shown in Figure 2D, chromatin clearance occurs in wild type mice but is strongly and significantly reduced in DNase1-deficient mice, showing a 55.1% increase of uncleared chromatin after 60 min, indicating that DNase1 is a key nuclease involved in elimination of circulating chromatin.

### Chromatin Activates Spleen Cells and Chromatin-Mediated Cell Activation Is Exacerbated in DNase1-Deficient Mice

**Figure 3:**
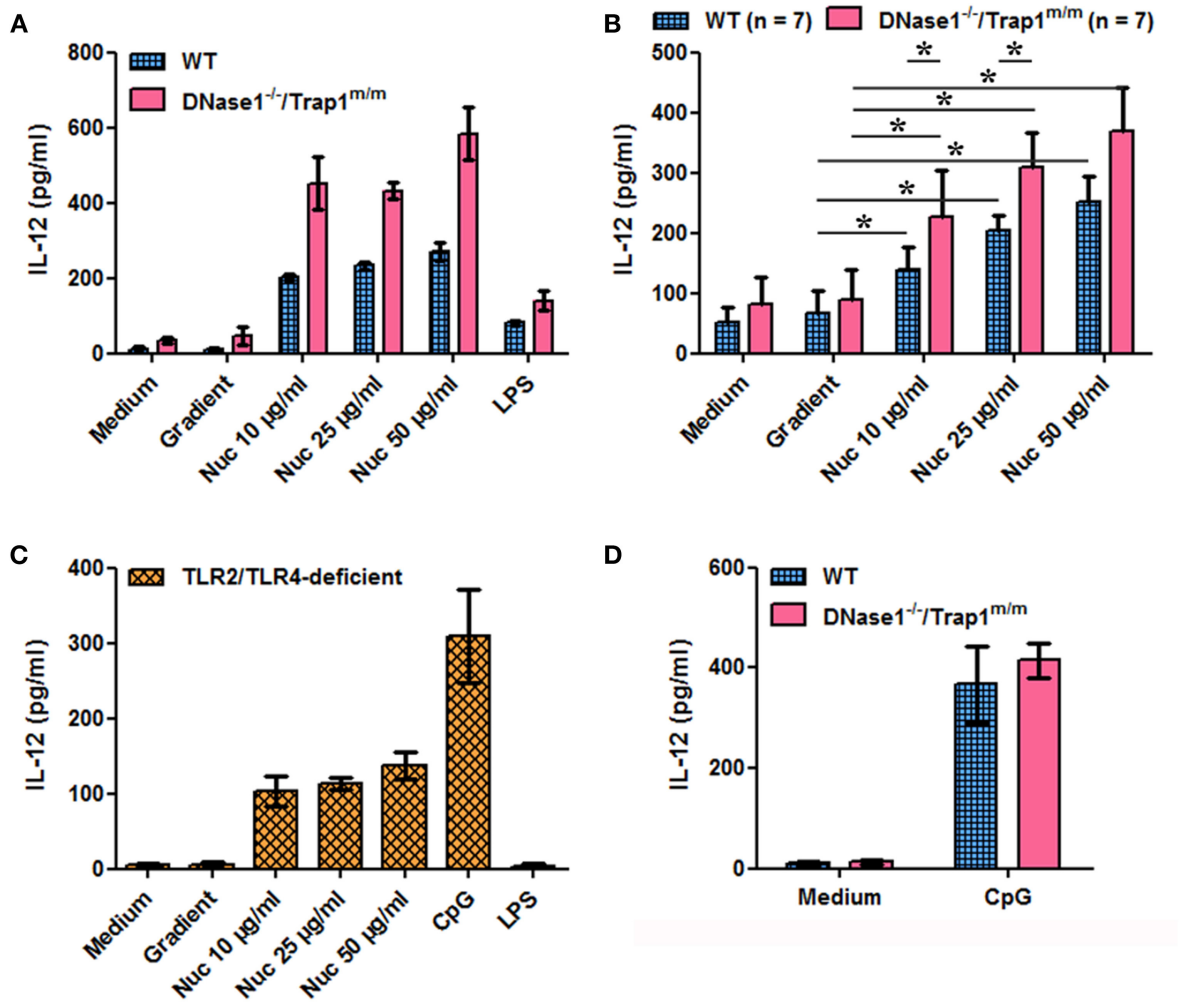
Nucleosome-induced IL-12 secretion is increased in spleen cells of DNase1-deficient mice. Spleen cells from wild type (WT), DNase1-deficient mice (*DNase1*^−/−^*/Trap1*^*m*/*m*^) and TLR2/TLR4-deficient mice were cultured in the presence/absence of different concentrations of purified nucleosomes (Nuc), the nucleosome purification buffer (gradient), LPS (TLR4 agonist) or CpG-ODN (TLR9 agonist). IL-12p40/p70 secretion was estimated by ELISA. **(A)** Shown is one representative experiment of seven independent experiments using four independent chromatin preparations. Means and SD of triplicates are depicted. **(B)** Data pooled from the seven independent experiments with seven mice/group are shown. Means and SEM of individual means are depicted. *p ≤ 0.05 (two-tailed Wilcoxon matched-pairs signed rank test). **(C)** Shown is one representative experiment of two independent experiments. Means and SD of triplicates are depicted. **(D)** Shown is one representative experiment of three independent experiments. Means and SD of triplicates are depicted.

**Figure 4:**
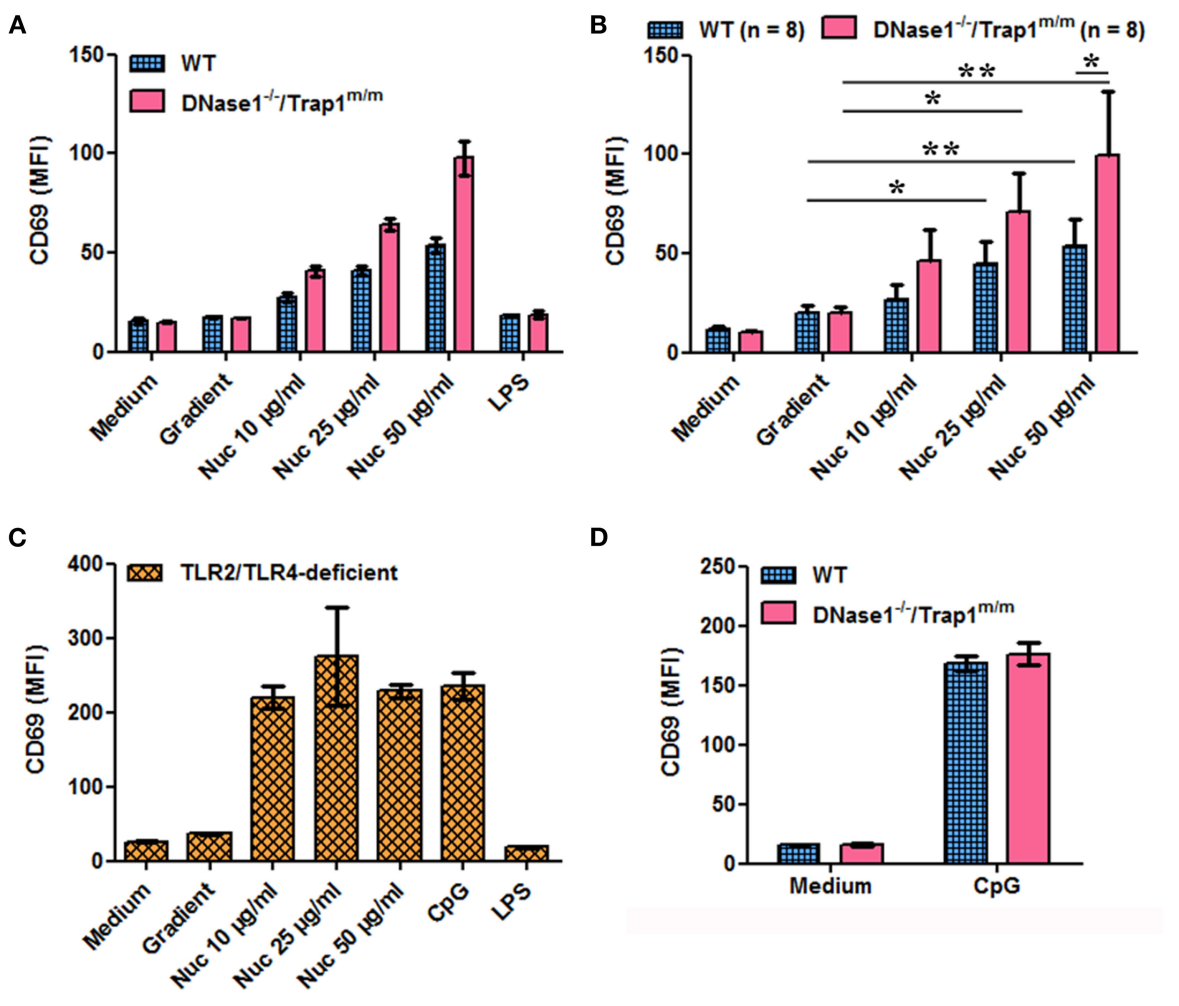
Nucleosome-induced CD69 expression is increased in spleen cells of DNase1-deficient mice. Spleen cells from wild type (WT), DNase1-deficient mice (*DNase1*^−/−^*/Trap1*^*m*/*m*^) and TLR2/TLR4-deficient mice were cultured in the presence/absence of different concentrations of purified nucleosomes (Nuc), the nucleosome purification buffer (gradient), LPS (TLR4 agonist) or CpG-ODN (TLR9 agonist). CD69 expression was estimated by flow cytometry. **(A)** Shown is one representative experiment of eight independent experiments using four independent chromatin preparations. Means and SD of triplicates are depicted. **(B)** Data pooled from the eight independent experiments with eight mice/group are shown. Means and SEM of individual means are depicted. *p ≤ 0.05; **p ≤ 0.01 (two-tailed paired *t*-test or two-tailed Wilcoxon matched-pairs signed rank test). **(C)** Shown is one representative experiment of two independent experiments. Means and SD of triplicates are depicted. **(D)** Shown is one representative experiment of three independent experiments. Means and SD of triplicates are depicted. MFI, mean fluorescence intensity. Cells were gated on total living cells according to size and granularity and exclusion of dead (propidium iodide-positive) cells.

We next investigated the potential consequences of impaired chromatin clearance. The data presented above show that cell-free chromatin is degraded in serum *in vitro* and in plasma *in vivo*, leading to mono-nucleosome accumulation before complete degradation, and that this process is delayed in the absence of active DNase1. Moreover, circulating chromatin has been shown to deposit in the spleen of mice *in vivo* (30). Yet, we have previously shown that extracellular nucleosomes are pro-inflammatory and behave like a DAMP, leading to neutrophil and dendritic cell activation in mice and humans, both in healthy individuals and lupus patients. To mimic an excess of circulating nucleosomes and to examine its potential consequences, we have stimulated spleen cells *in vitro* with purified nucleosomes, as spleens contain immune cells activated *in vivo* in patients with SLE. Nucleosomes activate spleen cells in a dose-dependent manner as estimated by IL-12 secretion (Figures 3A,B) and CD69 upregulation (Figures 4A,B). Both wild type and *DNase1*^−/−^*/Trap1*^*m*/*m*^ spleen cells respond to nucleosomes but interestingly cell activation was significantly enhanced in spleen cells from *DNase1*^−/−^*/Trap1*^*m*/*m*^ mice, both for IL-12 secretion (Figure 3B) and CD69 expression (Figure 4B). Regarding CD69 expression, not only the mean fluorescence intensity increased in a dose-dependent manner in response to nucleosomes, but also the percentage of CD69-positive cells, which was also higher in cells from *DNase1*^−/−^*/Trap1*^*m*/*m*^ mice than wild type mice. In contrast, LPS (a TLR4 agonist) strongly activated spleen cells, but the response was not significantly different between both mouse groups (Supplementary Figure 2). The observed differences in spleen cell activation are not due to an altered cell composition of spleens from *DNase1*^−/−^*/Trap1*^*m*/*m*^ mice, since we did not observe major or significant differences in the percentages of B and T lymphocytes, dendritic cells, neutrophils or activation levels estimated by CD86 expression between wild type and DNase1-deficient mice (Supplementary Figure 3). The viability of cells in culture is depicted in Supplementary Figure 4. We excluded that cell activation is mediated by endotoxin contamination of nucleosomes, as spleen cells from TLR2/TLR4-deficient mice are activated by nucleosomes [and CpG motif-containing oligonucleotides (ODN), a TLR9 agonist used as a positive control], but not by LPS (Figures 3C, 4C). Although LPS is not optimal for inducing some of the parameters analyzed, spleen cells strongly respond to CpG-ODN and importantly spleen cells isolated from both wild type and *DNase1*^−/−^*/Trap1*^*m*/*m*^ mice respond to the same extent (Figures 3D, 4D), excluding an intrinsic higher response to stimulation of cells from *DNase1*^−/−^*/Trap1*^*m*/*m*^ mice.

### Trap1 Expression Is Down-Regulated by Cytokines

**Figure 5:**
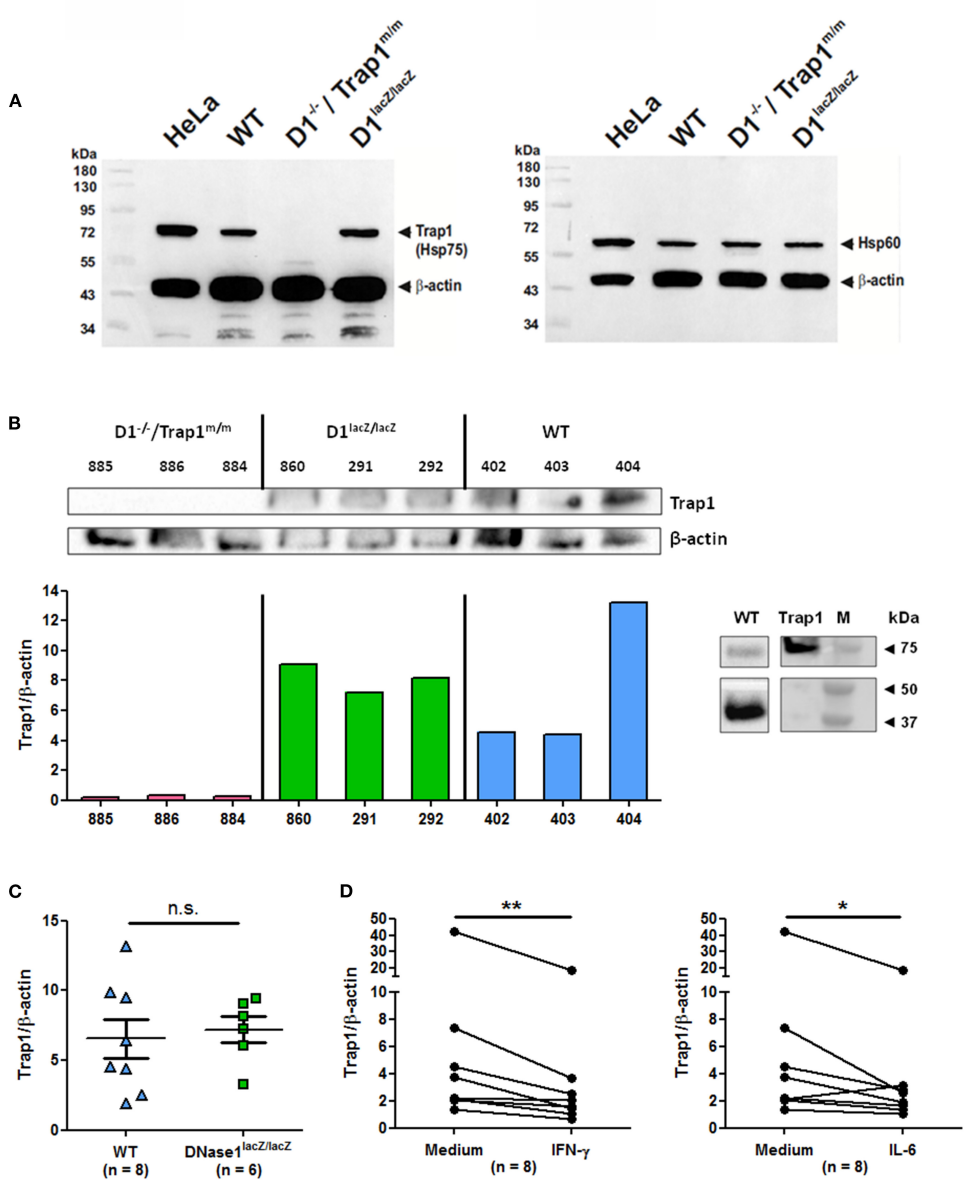
Modulation of Trap1 expression by cytokines. **(A)** Trap1 (Hsp75) deficiency in *DNase1*^−/−^*/Trap1*^*m*/*m*^ mice. Immunodetection by Western blot of mitochondrial Trap1 (Hsp75, ~74 kDa, left panel) and mitochondrial Hsp60 (~58 kDa, right panel) using splenocyte protein extracts (50 μg) of the mouse strains indicated. For loading control and for positive control, β-actin (~42 kDa) and human HeLa cell extract (50 μg) were investigated in parallel. The first lane corresponds to molecular weight markers. One representative experiment of two is shown. WT, wild type; D1^−/–^/Trap1^m/m^, DNase1^−/–^/Trap1^m/m^; D1^lacZ/lacZ^, DNase1^lacZ/lacZ^; kDa, kiloDalton. **(B)** Comparative analysis (left) of *ex vivo* Trap1 and β-actin expression in a series of splenocytes from the three mouse strains as in **(A)** with 25 μg protein extracts (top) and quantification of Trap1 expression after normalization to β-actin expression (bottom). Numbers indicate individual mice. Three mice were analyzed per genotype. As a positive control (right panel), recombinant Trap1 was loaded in parallel to protein extract from a wild type mouse and Trap1 (top) and β-actin (bottom) were immunodetected (lanes were extracted from the same Western blot but at different chemiluminescence exposure times). M, molecular weight markers. One representative experiment of two is shown. **(C)** Pooled data of Trap1 expression normalized to β-actin expression in splenocytes from eight independent wild type vs. six *DNase1*^*lacZ*/*lacZ*^ mice (25 μg protein extract were loaded for each mouse). n.s., not significant. Mean and SEM are shown. **(D)** *In vitro* down-regulation of Trap1 expression by cytokines. Splenocytes where cultured for 24 h in the presence/absence of IFN-γ (left) or IL-6 (right) and Trap1 expression was determined as in **(C)**. Shown are pooled data from three independent experiments with splenocytes from eight independent mice. **p < 0.01; *p ≤ 0.05 (two-tailed Wilcoxon matched-pairs signed rank test).

To correlate these data to Trap1 expression, we next analyzed *ex vivo* Trap1 expression by immunoblots, using Hela cells as a positive control, in spleen cells of the two mouse strains used above and in addition of *DNase1*^*lacZ*/*lacZ*^ mice, which are DNase1-deficient but supposed to normally express Trap1. As expected, Trap1 is expressed in wild type cells but not in cells from *DNase1*^−/−^*/Trap1*^*m*/*m*^ mice (Figure 5A). In contrast, Trap1 was clearly detected in cells from *DNase1*^*lacZ*/*lacZ*^ mice. In contrast to Trap1, expression of Hsp60 (an additional mitochondrial chaperone) is not non-specifically influenced by the Trap1 mutation in *DNase1*^−/−^*/Trap1*^*m*/*m*^ mice as all mouse strains investigated show a comparable expression. Trap1 expression was next quantified after normalization to β-actin expression in a series of individual mice and compared between mouse strains. A representative experiment showing Trap1 and β-actin expression as well as Trap1/β-actin expression ratios is depicted in Figure 5B, whereas Figure 5C presents pooled data from wild type and *DNase1*^*lacZ*/*lacZ*^ mice. As a positive control, recombinant Trap1 was analyzed by Western blot in comparison to spleen cell protein extract from a wild type mouse (Figure 5B, right panel). No Trap1 expression was detected in *DNase1*^−/−^*/Trap1*^*m*/*m*^ cells, but was clearly detected in cells from all wild type and *DNase1*^*lacZ*/*lacZ*^ mice. Particularly, Trap1 expression was similar in wild type and DNase1-deficient *DNase1*^*lacZ*/*lacZ*^ mice. It has been previously suggested Trap1 expression is negatively influenced by DNase1 expression (26). However, DNase1 expression is not detectable in splenocytes (39). More importantly, we could show that both IFN-γ and IL-6, two cytokines involved in lupus pathogenesis, significantly down-regulated Trap1 expression in spleen cells after *in vitro* culture (Figure 5D), whereas no change was observed with IFN-α or IL-17A (Supplementary Figure 5). These results support the usefulness of *DNase1*^−/−^*/Trap1*^*m*/*m*^ mice in studies focusing on lupus pathogenesis.

### Trap1 Deficiency Amplifies Nucleosome-Induced Cell Activation

**Figure 6:**
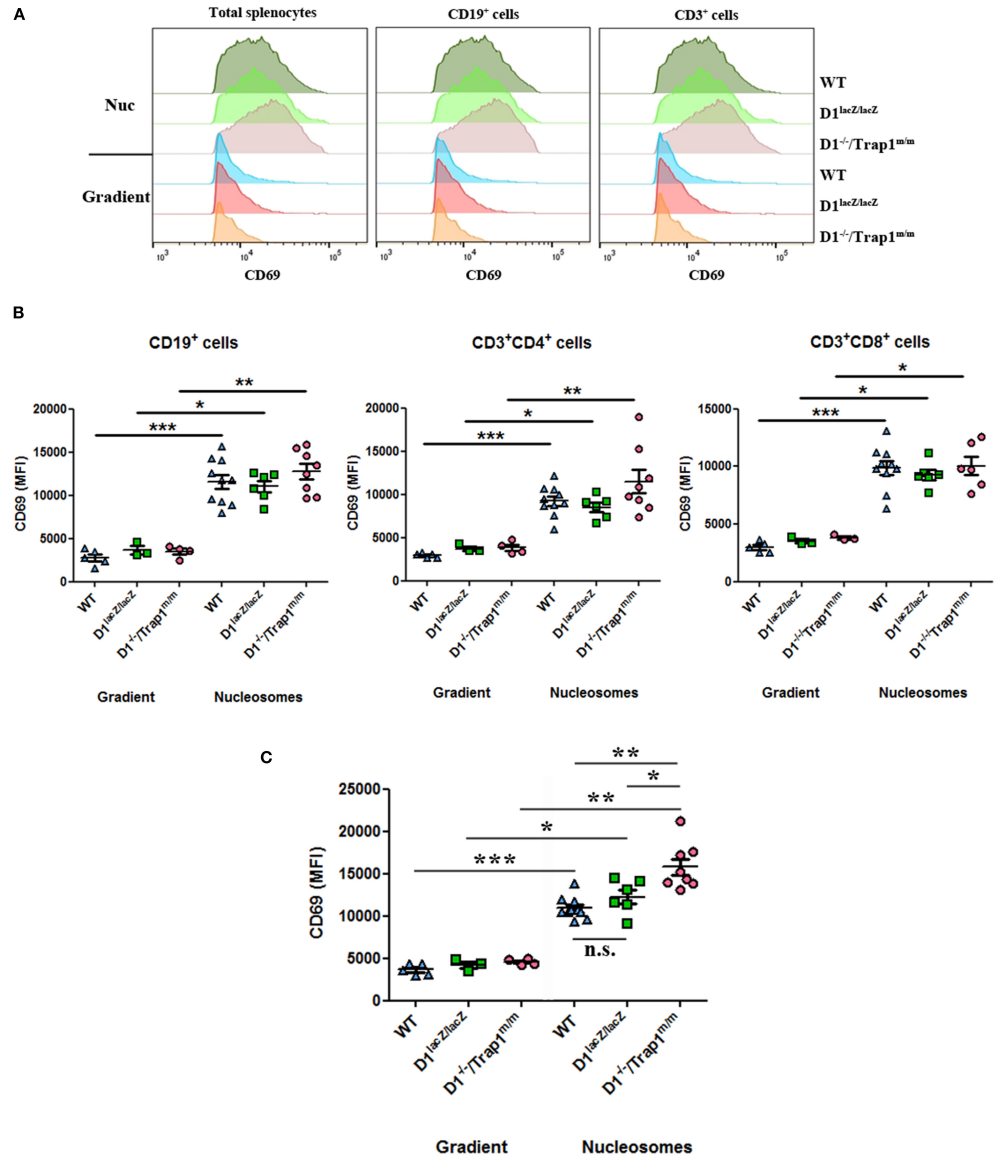
Trap1 deficiency explains enhanced nucleosome-induced cell activation in *DNase1*^−/−^*/Trap1*^*m*/*m*^ mice. Spleen cells from wild type (WT), *DNase1*^*lacZ*/*lacZ*^ (D1^lacZ/lacZ^) and *DNase1*^−/−^*/Trap1*^*m*/*m*^ (D1^−/–^/Trap1^m/m^) mice were cultured with nucleosomes (Nuc) or its purification buffer (gradient) and CD69 expression was analyzed by flow cytometry on total splenocytes or after gating on CD19^+^, CD3^+^, CD3^+^CD4^+^, or CD3^+^CD8^+^ cells. **(A)** CD69 histograms for a representative mouse of each genotype. **(B,C)** Data pooled from all mice analyzed in two independent experiments and using two independent chromatin preparations (**B**, lymphocyte sub-populations; **C**, total spleen cells). MFI, mean fluorescence intensity; n.s., not significant. *p < 0.05; **p < 0.01; ***p < 0.001 (two-tailed unpaired *t*-test with Welch's correction or two-tailed Mann Whitney test). Mean and SEM are shown.

Our data show that absence of serum/plasma DNase1 leads to an increased concentration of circulating nucleosomes, which in turn may activate immune cells upon deposition in spleens, especially in DNase1-deficient mice. Because an intrinsic DNase1 expression and activity are not detectable in wild type mouse spleen by zymography (39) and because we observed that Trap1 expression is similar in *DNase1*^*lacZ*/*lacZ*^ and wild type mice (Figure 5C), we compared activation of splenocytes from these three mouse strains after *in vitro* exposure to nucleosomes. A significant nucleosome-induced activation (estimated by CD69 expression) was observed with total spleen cells of all mouse strains (Figure 6A, left and Figure 6C). CD69 was also significantly up-regulated by nucleosomes in comparison to the purification buffer on both CD19^+^ and CD3^+^ cells from the three individual mouse strains (Figure 6A, middle and right, and Figure 6B), and among T lymphocytes on both CD4^+^ and CD8^+^ cells (Figure 6B). As a control, no difference was observed for CD69 expression with the purification buffer between the three genotypes. However, whereas activation was similar in wild type and *DNase1*^*lacZ*/*lacZ*^ mice, cell activation was significantly enhanced in splenocytes from *DNase1*^−/−^*/Trap1*^*m*/*m*^ mice in comparison to both wild type and *DNase1*^*lacZ*/*lacZ*^ splenocytes (Figure 6C). Nevertheless, when the response to nucleosomes was compared between mouse strains, CD69 expression was only significantly enhanced on total splenocytes from *DNase1*^−/−^*/Trap1*^*m*/*m*^ mice in comparison to both wild type and *DNase1*^*lacZ*/*lacZ*^ mice (Figure 6C), but did not reach statistical significance on CD19^+^ and CD3^+^ cells (Figure 6B). Altogether, the results indicate that Trap1 deficiency is responsible for the exacerbated nucleosome-induced spleen cell activation in *DNase1*^−/−^*/Trap1*^*m*/*m*^ mice.

## Discussion

DNase1 is a major serum nuclease. Here we show that DNase1 activity plays a key role in the degradation of extracellular chromatin *in vitro* in serum and in the clearance of circulating chromatin *in vivo* in plasma. DNase1-mediated chromatin degradation does not require C1q, is crucial when DNase1-like 3 activity is impaired and is particularly efficient after activation of the plasminogen system. Inactivation of DNase1 leads to delayed elimination of chromatin *in vivo* and accumulation of mono-nucleosomes, which have a known pro-inflammatory activity. Indeed, we demonstrate that mono-nucleosomes activate spleen leukocytes in a concentration-dependent manner. Moreover, nucleosome-induced cell activation is enhanced in the absence of Trap1. Finally, we also report that some lupus-associated cytokines down-regulate Trap1 expression.

As previously reported (34), serum of wild type animals does digest chromatin without heparin, as evidenced by the accumulation of mono-nucleosomes after 4–8 h and DNase1-like 3 is probably also involved. In the present study, the level of degradation is lower than in the above mentioned study because we used a much higher chromatin concentration, and we analyzed degradation at 4 h instead of 8 h. Moreover, we used here purified chromatin instead of isolated nuclei, which may influence the degradation rate. In nuclei, chromatin is associated with additional proteins and is organized in higher compacted structures, influencing DNase1 accessibility.

Several aspects are however novel compared to Napirei et al. (34) and our data extend the latter study. Firstly, as mentioned above, we used purified chromatin (which is the natural circulating material) instead of isolated nuclei. Secondly, we analyzed the influence of C1q deficiency, alone or in combination with DNase1 deficiency. Moreover, we investigated the *in vivo* chromatin degradation in addition to *in vitro* assays. Likewise, we determined the consequences of impaired chromatin degradation on immune cell activation. Finally, the present manuscript shows and explains how a combined DNase1/Trap1 deficiency might be deleterious in SLE.

Using a different approach, no difference in circulating DNA levels was observed between wild type and DNase1-deficient mice (40). This apparent discrepancy might be partly explained by the young age of mice. Moreover, a kinetic study over several weeks may be more informative. Likewise, the data presented focused on mono-nucleosome-like particles; our approach allows the analysis of chromatin fragments of different sizes, which gives additional information. Finally, the latter study analyzed the natural load of chromatin in the circulation, whereas we investigated an overload of chromatin.

Recently, a different strain of DNase1-deficient mice with an intact *Trap1* gene has been shown to develop a lupus-like disease on a pure C57BL/6 genetic background (23). The DNase1/Trap1-KO mice used in the present study do not develop lupus on the pure C57BL/6 genetic background (13), but develop the disease on the mixed 129 × C57BL/6 F2 genetic background (21), indicating that other genetic factors contribute to lupus. Several reasons may explain the differences in lupus development in the two studies. First of all, Kenny et al. analyzed mice at later time points (anti-nuclear antibodies and anti-nucleosome antibodies were measured at 9 months of age vs. 8 months by Napirei et al., whereas glomerulonephritis was evaluated at 12 vs. 8 months). There are also differences in the methods used for anti-nuclear antibody detection. Napirei et al. detected anti-nuclear antibodies by immunofluorescence using fixed culture cells (to evaluate the nuclear staining pattern), whereas Kenny et al. used ELISA which is probably more sensitive.

It may be interesting to analyze whether DNase1 and Trap1 contribute together to aberrant immune activation *in vivo* and lupus development. This was not the aim of the present study and would require a different approach. For that purpose, Kenny's mice could be backcrossed on a mixed 129 × C57BL/6 genetic background, or Kenny's and Napirei's mice should be intercrossed to generate DNase1-KO and DNase1/Trap1-double KO mice with the same genetic background. In addition, mouse strains should be compared in the same facility and under the same housing conditions, as environmental factors are known to contribute to autoimmunity development.

SLE is a systemic autoimmune disease with skin, joint, renal, cardiovascular and nervous system manifestations. The disease is probably declared when a combination of genetic and environmental factors is encountered. A defect in apoptosis regulation (41) or apoptotic cell clearance (42) has also been suggested to be involved in the disease, resulting in secondary necrosis and the release of cell content into the circulation (43), creating a pro-inflammatory milieu (44). A combination of the latter events may result in the generation and release of nucleosomes in patients. More recent data suggest that part of circulating nucleosomes may derive from neutrophil extracellular traps (NET), especially from the smooth stretches (45). Activated PMN release NET, fibers composed of DNA and associated proteins (46). PMN are indeed activated in SLE (47, 48) and some SLE patients have an impaired capacity to clear NET due to impaired DNase1 function, which correlates with high anti-double-stranded DNA autoantibody titers and renal involvement (49). In addition, chromatin may be liberated by necrotic cells, after tissue damage, and from different cell types, before being processed in the extracellular milieu. Chromatin derived from necrotic cells is recognized by Clec2d, which triggers a pro-inflammatory response (50). The origin of circulating nucleosomes might be determined by analyzing nucleosome positioning (51).

Several lines of evidence support the pathogenic role of circulating nucleosomes in SLE. First, levels of circulating chromatin are correlated to disease activity (3). Beside anti-double-stranded DNA autoantibodies, anti-H1 autoantibodies have been shown to be a highly specific marker for SLE and are associated with increased disease activity (52). Renal involvement represents a severe manifestation in SLE. Glomerulonephritis is due to an inflammatory response caused by immune complex deposition in kidney glomeruli followed by activation of the complement system, resulting in tissue damage in the form of a type III hypersensitivity reaction. Importantly, immune complexes composed of anti-nucleosome autoantibodies are believed to be pathogenic upon deposition in kidneys. This process leads to the production of the C5a complement factor, which is known to act as a chemotactic factor for PMN. Nucleosomes have been detected in glomerular deposits in human lupus nephritis (53). Likewise, nucleosomal antigens have been found in the epidermal basement membrane of nonlesional skin of patients with lupus nephritis (54). These results suggest that deposited nucleosomes mediate the binding of autoantibodies in tissues, leading to inflammation. Of note, immune complex deposition and inflammation also occur in joints and vessel walls of lupus patients, resulting in arthritis and general vasculitis, respectively. Thus, in addition to their DAMP activity, nucleosomes may be pathogenic via *in situ* immune complex formation.

We analyzed the consequences of increased concentrations of extracellular nucleosomes focusing on splenocytes. Indeed, in addition to kidneys, intravenously injected nucleosomes deposit in the spleen (30). Moreover, the spleen contains immune cells known to be activated in SLE and especially some of them are directly activated by nucleosomes, e.g., dendritic cells (6) and neutrophils (7), even *in vivo*. In addition, nucleosomes interact with mouse spleen cells to induce cytokine secretion and polyclonal B-cell activation (55, 56). We thus assumed that chromatin deposition is increased in DNase1-deficient mice *in vivo* and we used *in vitro* culture to mimic this mechanism. We could show that extracellular mono-nucleosomes significantly activate spleen leukocytes. Both B and T lymphocytes were activated, and among T lymphocytes both CD4^+^ and CD8^+^ cells, although we could not characterize in more detail the activated cell population. Likewise, to estimate cell activation, we also analyzed CD86 and class II MHC expression, as well as IL-6 secretion, but no difference between mouse groups was observed. CD69 was used here as an activation marker but its precise biological significance in this context is unclear. We also show for the first time that nucleosome-induced activation is exacerbated in *DNase1*^−/−^*/Trap1*^*m*/*m*^ mice, as a result of Trap1 deficiency, since DNase1 is not expressed in the spleen and because no difference between wild type and DNase1-deficient mice without Trap1 impairment was found. Interestingly, in addition to the transient *Trap1* down-regulation in end-stage lupus disease (26) and *Trap1* gene polymorphisms associated with susceptibility to SLE (27), *Trap1* mutations have been linked to autoinflammation and impaired Trap1 function has been linked to increased cellular stress and elevated serum IL-18 concentrations (57). These results suggest a protective role for Trap1. Actually, we have previously reported that nucleosomes induce oxidative burst in PMN (10). Further experiments will be required to determine whether nucleosomes induce oxidative stress in splenocytes and whether this stress mediates cell activation. Likewise, the pathway triggered by nucleosomes and the receptor involved are unknown. However, we have previously shown that activation of immune cells by free nucleosomes occurs independently of MyD88 (6) and independently of TLR9 (9), a typical receptor for DNA. Activation of immune cells through TLR9 mainly occurs when nucleosomes are in immune complexes (58, 59) upon internalization via Fc receptors. This is the reason why nucleosomes, but not CpG-ODN, differently activate spleen cells from wild type and DNase1/Trap1-deficient mice, which both express TLR9. This implies that Trap1 may partly control the signal triggered by nucleosomes but not CpG.

Finally, we demonstrate that lupus cytokines down-regulate Trap1 expression. The fact that IFN-γ and IL-6 significantly down-regulated Trap1 *in vitro* suggests that those cytokines or other lupus-associated cytokines may similarly influence *Trap1* expression in patients, potentially not exclusively in the spleen. Several studies have suggested that these two cytokines are implicated in lupus pathogenesis, both in patients and mouse models. Nephritis appears to be particularly dependent on IFN-γ in mice (60). Likewise, IL-6 blockade ameliorates lupus in mice and prevents anti-DNA autoantibody production (61) whereas IL-6 concentration is elevated in SLE patients (62). In addition, these two cytokines are atherogenic in SLE (63). However, we did not observe Trap1 down-regulation by the lupus cytokine IFN-α. Although IFN-α is an important lupus cytokine, other cytokines are clearly involved in lupus pathogenesis. These results suggest that all lupus cytokines do not impact Trap1 expression, although we do not have a clear explanation. We believe that different combinations of cytokines are probably involved in modulating Trap1 expression and this hypothesis should be tested. In agreement with our results, TNF (which is secreted at higher levels in SLE patients) has been shown to reduce Trap1 protein expression in renal cells (64).

Although our data suggest that impaired DNase1 activity is pathogenic in SLE, other factors (alone or in combination with DNase1) may lead to accumulation of circulating chromatin and anti-chromatin autoimmunity. The role of DNase1 in nucleosome degradation *in vitro* was previously reported, using recombinant DNase1 instead of serum (65). These factors may be involved in the degradation of chromatin released in the extracellular milieu or, at an earlier step, in the clearance of apoptotic cells. Thus, mice deficient for the serum nuclease DNase1-like 3 develop anti-double-stranded DNA autoantibodies via a mechanism involving extrafollicular B-cell differentiation into short-lived antibody-forming cells (66). DNase1-like 3 deficiency leads to aberrations in the fragmentation of plasma DNA, e.g., an increase in short DNA molecules (67). Cell-free DNA fragmentation actually depends on both intracellular and extracellular nucleases (68). Deficient clearance may result from deficiency in the complement system. Indeed, complement proteins mediate the clearance of apoptotic cells by macrophages (69) and C1q is particularly important for the clearance of apoptotic cells *in vitro* (70) and *in vivo* (71). Actually, C1q binds to apoptotic cells (72). Consistently, serum C1q concentrations are decreased in SLE patients (73) and C1q-deficient mice develop a lupus-like disease (31). Finally, extracellular nucleosomes inhibit phagocytosis of apoptotic cells by macrophages of lupus mice (74), potentially leading to an amplification loop.

In conclusion, plasma chromatin degradation requires DNase1 and is more efficient when the plasminogen system is activated. Indeed, in SLE both a lower DNase1 activity and a reduced fibrinolytic activity have been reported. Chromatin degradation by DNase1 is also efficient when DNase1-like 3 activity is low, as observed in SLE patients. A transient low Trap1 expression may increase the pathogenic role of extracellular nucleosomes. This combination represents a new mechanism regulating the clearance and the activity of a DAMP; it may favor the break of peripheral tolerance and the triggering of anti-nucleosome autoimmunity as a result of defective clearance of nucleosome, which might partly explain SLE development in genetically predisposed individuals.

## Data Availability Statement

The raw data supporting the conclusions of this article will be made available by the authors, without undue reservation.

## Ethics Statement

The animal study was reviewed and approved by Regierungspräsidium Tübingen, reference IM4/07 and the Darwin Committee of the University Sorbonne Paris Nord.

## Author Contributions

PD designed the research, performed part of the experiments, coordinated the study, analyzed and interpreted data, and wrote the manuscript. JF and AE performed the experiments and analyzed and interpreted data. MB, RH, and DL performed the experiments. HM analyzed the data. MN performed part of the experiments and analyzed and interpreted data. H-GR analyzed and interpreted data. All authors contributed to the article and approved the submitted version.

## Conflict of Interest

The authors declare that the research was conducted in the absence of any commercial or financial relationships that could be construed as a potential conflict of interest.
