# Supplementary material for: Deoxyribonuclease 1-Mediated Clearance of Circulating Chromatin Prevents From Immune Cell Activation and Pro-inflammatory Cytokine Production, a Phenomenon Amplified by Low Trap1 Activity: Consequences for Systemic Lupus Erythematosus
Source: Front Immunol. 2021 Mar 4;12:613597. doi: 10.3389/fimmu.2021.613597 (PMC7969502; doi:10.3389/fimmu.2021.613597)
Supplement: Supplementary file 1 [file Data_Sheet_1.pdf]

Supplementary Material

Supplementary Figures

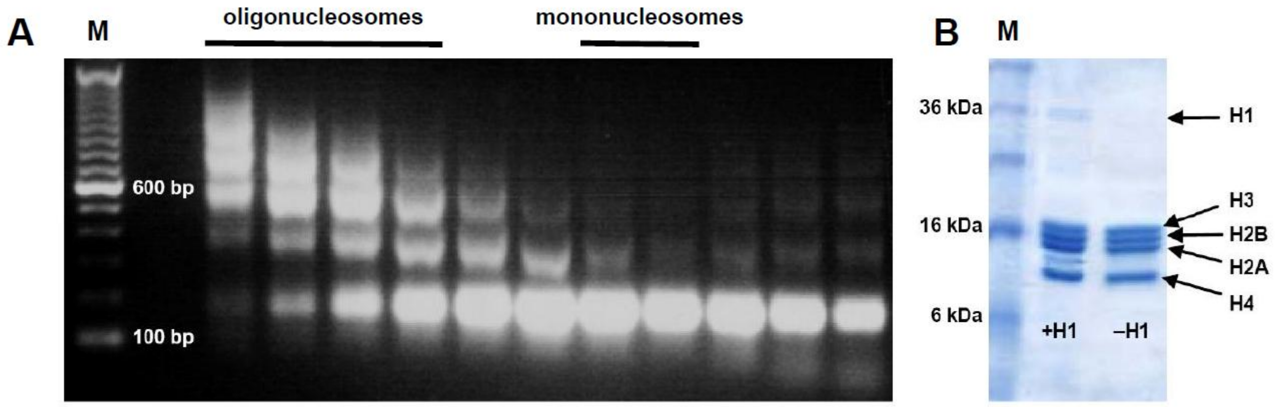

Supplementary Figure 1. Representative preparations of chromatin yielding mono-nucleosomes and oligo-nucleosomes. Fractions collected after ultracentrifugation were characterized on an 1.5 % agarose gel (A) and an 18 % SDS-PAGE (B). In (A), fractions used as mono-nucleosomes and oligo-nucleosomes are marked. In (B), preparations incubated without or with NaCl and thus containing histone H1 or not, respectively, are depicted. bp, base pairs; kDa, kiloDaltons; H, histone; M, molecular weight marker. The doublet at ~ 35 kDa (B) represents histone H1. Shown is one representative of eight independent experiments.

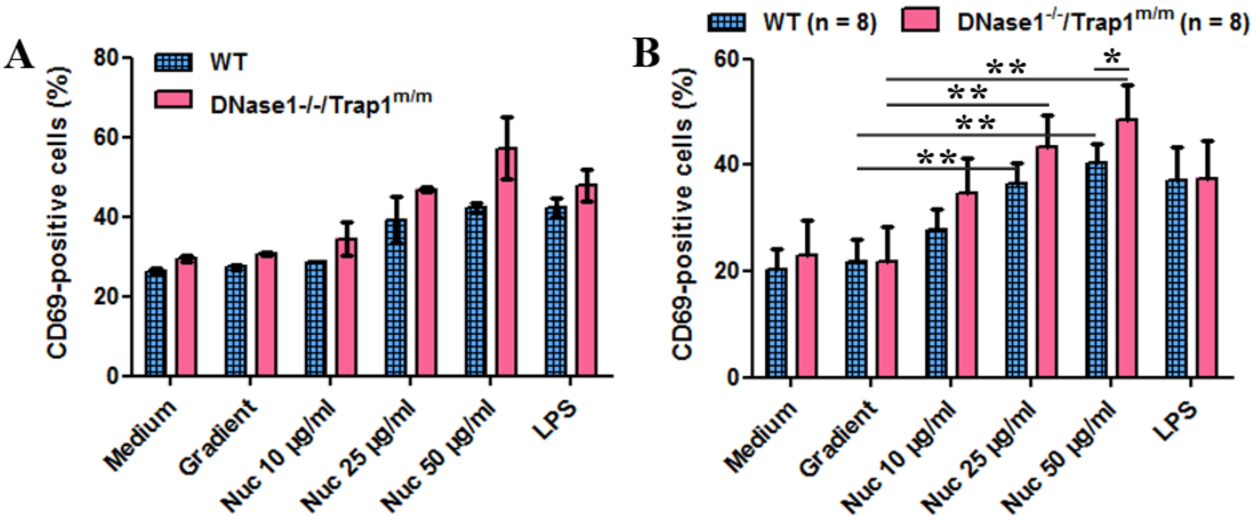

Supplementary Figure 2. Nucleosome-induced CD69 expression is increased in spleen cells of *DNase1$^{-/-}$/Trap1$^{m/m}$* mice. Spleen cells from wild type (WT) and DNase1-deficient mice (*DNase1$^{-/-}$/Trap1$^{m/m}$*) were cultured in the presence/absence of different concentrations of purified nucleosomes (Nuc), the nucleosome purification buffer (gradient) or LPS (a TLR4 agonist). CD69 expression was estimated by flow cytometry and is presented as percentages of CD69-positive cells among all spleen cells. Cells were gated on total living cells according to size and granularity and exclusion of dead (propidium iodide-positive) cells. **(A)** Shown is one representative experiment of eight independent experiments with four independent chromatin preparations. Means and SD of triplicates are depicted. **(B)** Data pooled from the eight independent experiments with eight mice/group are shown. Means and SEM of individual means are depicted. *, $p \leq 0.05$; **, $p \leq 0.01$ (two-tailed paired *t*-test or two-tailed Wilcoxon matched-pairs signed rank test).

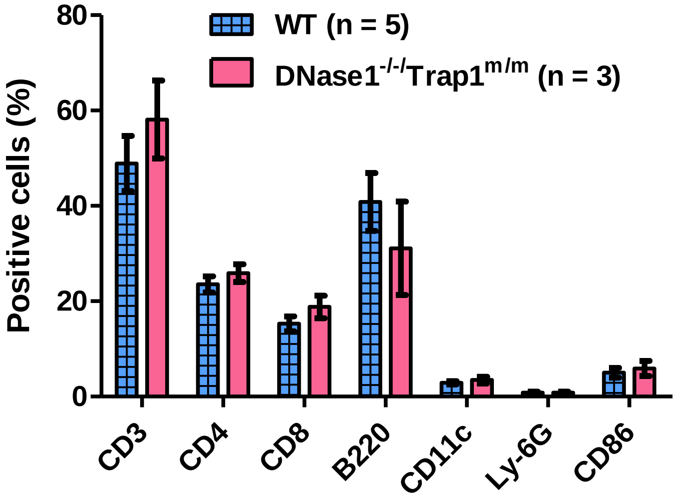

Supplementary Figure 3. Cell composition of spleens from wild type and *DNase1$^{-/-}$/Trap1$^{m/m}$* mice. Freshly isolated spleen cells from wild type (WT, n = 5) and *DNase1$^{-/-}$/Trap1$^{m/m}$* (n = 3) mice were stained ex vivo for plasma membrane CD3, CD4, CD8, B220, CD11c, Ly-6G and CD86 and analyzed by flow cytometry. The graph depicts percentage of cells positive for the indicated marker among total live spleen cells gated as in supplementary Figure 2. Shown are pooled data from three independent experiments. Mean and SEM are depicted.

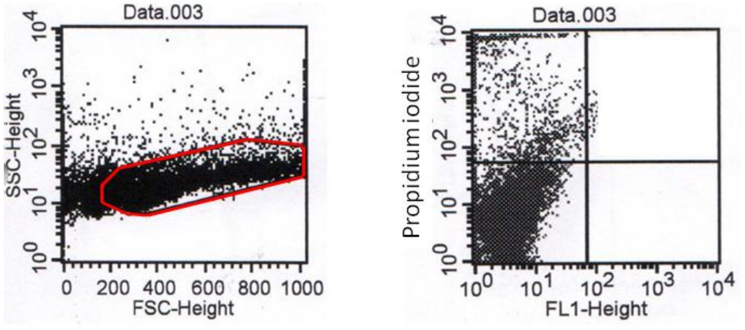

R1 = 60 %

PI = 18 %

Supplementary Figure 4. Viability of spleen cells in culture. Freshly isolated spleen cells were cultured for 65 hours in X-VIVO 15 medium without serum and were analyzed by flow cytometry. The left panel depicts the gate (R1 = 60 %, in red), according to size (FSC) and granularity (SSC), used to analyze cell activation (after staining with monoclonal antibodies specific for cell surface markers). The right panel shows the overall mortality after propidium iodide (PI) staining on total cells (not R1-gated), which represents 18 % of dead cells among total spleen cells, representing less than 5 % of dead cells in R1-gated cells. Shown is one representative experiment of eight independent experiments.

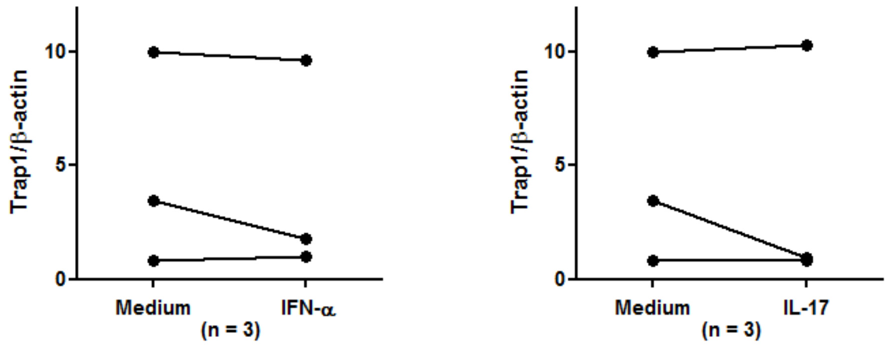

Supplementary Figure 5. No clear modulation of Trap1 expression by IFN-α or IL-17A. Splenocytes where cultured for 24 hours in the presence/absence of IFN-α (left) or IL-17A (right) and *in vitro* Trap1 expression was determined as in Figure 5D. Shown are pooled data from three independent experiments with splenocytes from three different mice.
